# Development of the SWB-HL: A Scale of the Subjective Well-Being of Older Adults With Hearing Loss

**DOI:** 10.3389/fpsyg.2021.640165

**Published:** 2021-06-11

**Authors:** Larry E. Humes

**Affiliations:** Department of Speech, Language, and Hearing Sciences, Indiana University, Bloomington, IN, United States

**Keywords:** hearing aid, quality of life, hearing health, aging, subjective well-being

## Abstract

The objective of this research was to develop and evaluate a self-report measure of subjective well-being (SWB) for use with older adults with hearing loss (HL). A convenience sample of 173 local volunteers between the ages of 60 and 88 years (*M* = 74.4; SD = 7.2 years) participated in this study. The initial 18-item version of the scale was constructed, response characteristics examined, and then subjected to factor analysis, as well as evaluation of the scales' reliability and validity. The analysis of response characteristics and subsequent factor analysis resulted in the elimination of eight of the 18 test items. The SWB-HL Total score was derived from the 10 remaining items. It was shown that the SWB-HL tapped three underlying domains interpreted as: Life Satisfaction (three items); Acceptance of Hearing Loss (Accept HL; four items); and Social Support (three items). Psychometric analysis showed very good reliability and good criterion validity was established for the 10-item SWB-HL Total score. In addition, significant differences were observed between aided and unaided SWB-HL Total scores following 4–6 weeks of hearing aid use. The SWB-HL is a 10-item self-report measure of SWB that shows good reliability and validity when used by older adults with hearing loss and reveals improved SWB following the use of hearing aids.

## Introduction

Recognizing the burgeoning population of older adults worldwide and the importance of facilitating healthy aging, on December 14, 2020, the United Nations adopted a resolution declaring 2021–2030 as the “Decade of Healthy Aging” (United Nations, 2020). Central to heathy aging is the concept of “well-being” (World Health Organization, 2015). Measures of well-being include both objective and subjective measures. Objective measures may include the disabilities experienced, life experiences, such as marriage and divorce, and annual income, among others. Subjective measures are labeled as such because they are self-report measures provided by a respondent. It is the latter, subjective well-being (SWB), that is the focus of this report.

Since the early work of Diener (1984), SWB was conceptualized as having three primary components: (1) life satisfaction; (2) frequent positive affect; and (3) infrequent negative affect (Diener et al., 1999). There have been two general approaches to the measurement of SWB: (1) a cognitive, reflective approach which asks the respondent to think about their life over some period of time, typically days, weeks, or months, and make evaluative ratings of their life satisfaction and affect (Diener, 1984); and (2) application of more time-locked immediate ratings in naturalistic or every-day environments, such as the Experience Sampling Method (Scollon et al., 2003), Ecological Momentary Assessment (Stone et al., 1999), and the Day Reconstruction Method (Kahneman et al., 2004). Perhaps because of the ease of measurement and the subsequent data reduction, most measures used on a widespread basis have been of the cognitive, reflective type. In particular, the most widespread measures make use of 5- to 7-point Likert-scale ratings of various aspects of life satisfaction and happiness or affect. Of SWB measures of this type, it appears that the Satisfaction with Life Scale (Diener et al., 1985) and the Positive and Negative Affect Scale (PANAS; Watson et al., 1988) have been most widely used to capture the three primary components of SWB. More recently, the Scale of Positive and Negative Experience (SPANE; Diener et al., 2010) has been used often as an alternative to the PANAS to capture the affect components of SWB.

A variety of life events are known to impact SWB. The development of hearing loss with advancing age is one such life event, one most commonly occurring with gradual onset. Age-related hearing loss is found in 65% of individuals above 60 years of age worldwide and has significant negative impacts on quality of life or SWB when left untreated (World Health Organization, 2021). The negative impact of hearing loss in older adults on health-related quality of life has been observed whether the impact was measured with general SWB measures or hearing-specific SWB measures (e.g., Bess et al., 1989, 1991; Mulrow et al., 1990; Dalton et al., 2003; Abrams et al., 2005; Chia et al., 2007; Ciorba et al., 2012; Nordvik et al., 2019). On the other hand, for the most part, benefits from intervention with hearing aids for older adults have been easy to document only on hearing-specific measures of SWB (e.g., Bess, 2000; Chisolm et al., 2005, 2007; McArdle et al., 2005; Dawes et al., 2015; Weinstein et al., 2016). The more that the items of the measure were specific to hearing loss, the more likely benefits could be observed. This could lead one to conclude that the benefits of intervention are limited to those within the hearing domain rather than impacting general SWB.

Perhaps a measure of SWB can be primed to be sensitive to the impact of hearing loss on SWB. Self-report surveys in general reveal broad effects of question context (Bertrand and Mullainathan, 2001; Lucas, 2018) and the operation of specific mechanisms such as priming (e.g., Bowling and Windsor, 2008; Garbarski et al., 2015). Similar effects of context and priming have been observed in the measurement of SWB as well (e.g., Schwarz and Strack, 1991; Kahneman et al., 1993). If each type of query is embedded within the same instrument, perhaps the net effect will be to capture variance shared by both hearing-specific and general SWB (e.g., Gehlbach and Barge, 2012). That is, by including queries about hearing loss within a broader measure of SWB can that measure of SWB be primed to be sensitive to the impact of hearing-aid intervention on SWB? Schwarz and Strack (1991) provided an interesting demonstration illustrating possible priming effects on SWB measures using two queries with young adults, one about the frequency of dating and one about life happiness. The responses to each query were moderately correlated (*r* = 0.66) when asked first about dating frequency but uncorrelated (*r* = −0.12) when the order was reversed. When another group of respondents was informed that queries would be made about “two areas of life that may be important to overall well-being,” the responses were again uncorrelated (*r* = 0.15). This suggests that measures of SWB which incorporate queries about hearing loss will attune the respondent to the importance of hearing loss when responding to queries about their general SWB.

Several years ago, unaware of any existing survey that blended both hearing-related queries and general SWB items together, an internet-search was conducted for such measures for other disorders or treatments. This search led to a series of tools made available by www.FACIT.org. The Functional Assessment of Chronic Illness Therapy (FACIT) organization “…manages the distribution and information regarding administration, scoring and interpretation of a range of questionnaires that measure health-related quality of life for people with chronic illnesses.” At the time of our search, one available measure seemed closest to our needs: Functional Assessment for Non-Life-Threatening Conditions (FANLTC). The FANLTC (Version 4) consisted of a total of 26 items, seven on physical well-being, seven covering social/family well-being, five dealing with emotional well-being, and seven concerned with functional well-being. The first seven items on physical well-being were deleted as they dealt with unlikely consequences of hearing loss including whether the respondent felt ill, had nausea, was bothered by treatment side effects, had pain, and so on. This scale on physical well-being reflects the roots of the FACIT organization's measures which began with the Functional Assessment of Cancer Therapy (FACT; Cella et al., 1993). As noted by Cella et al. (1993) regarding the FACT, for cancer patients undergoing treatment, the negative impacts of the treatment(s) on SWB often outweigh the impacts of the cancer being treated. For intervention with hearing aids, however, treatment side effects akin to those experienced during cancer therapy were not considered likely and the items pertaining to physical illness were dropped. In addition, one item in the social/family well-being section was deleted which inquired about the respondent's satisfaction with his or her sex life. This left a total of 18 items remaining. The other key change made to five of the remaining 18 items of the FANLTC involved changing all references to “my illness” to “my hearing loss.” The response options and instructions remained the same as for the FANLTC. The response options for each item (points assigned) were: not at all (0); a little bit (1); somewhat (2); quite a bit (3); and very much (4). The final version that emerged from these analyses is referred to as the Subjective Well-Being of Older Adults with Hearing Loss (SWB-HL) and will be described in more detail below.

The SWB-HL was administered to a group of 173 older adults prior to being fit with amplification and again as an aided measure for 143 of the 173 who received hearing aids in this study. In addition to the SWB-HL, several other measures were obtained prior to being fit with amplification, ranging from general measures of depression, anxiety, and optimism to detailed assessment of the communication difficulties experienced by these older adults and their adjustment to these difficulties. These measures will be used here to assess the validity of the SWB-HL. In addition to examining the validity of the SWB-HL, analyses were performed to evaluate the reliability of the SWB-HL and its subscales. Classical test theory was used to assess reliability due, in part, to the sample size being insufficient for so-called modern psychometric analysis, such as item response theory and Rasch analysis, which have been applied to audiological self-report measures in recent years (Heffernan et al., 2019; Cassarly et al., 2020). Finally, as another assessment of the validity of the SWB-HL and its sensitivity to change in SWB following hearing-aid use, we compare the aided and unaided SWB-HL scores of the older adults fit in this study.

## Materials and Methods

### Participants

A total of 173 older adults were recruited into the university clinic through advertisements in the newspaper, community centers, religious centers, and other facilities likely to be frequented by older adults in the Bloomington, Indiana community. The ads made it clear that the study was seeking those interested in hearing aids. In addition, some participants were recruited into the study from the clinic. That is, they came to the clinic on their own accord and once they completed the hearing evaluation, they were approached about participation in the study. As a result, this sample is a clinical convenience sample of older adults from the local community.

From 2004–2008, there were a total of 530 individuals who were screened for study eligibility. Of these, 162 (30.6%) were ineligible. Of the remaining 388 eligible individuals, 154 (39.7%) enrolled and purchased hearing aids with 143 completing the measures included in these analyses. They paid the full purchase price for the devices at the time of enrollment. Of the 234 eligible candidates who opted not to purchase hearing aids and enroll, 36 agreed to return to complete several of the pre-fit measures completed by the hearing-aid purchasers with 30 of these individuals completing all the measures included in these analyses. The total sample for these analyses was 173, including 143 individuals who were fitted with hearing aids and 30 who opted not to pursue hearing aids.

Of the 173 participants, 102 (59.0%) were male, 65 (37.6%) were female, and 6 (3.5%) did not indicate their gender. Participants ranged in age from 60 to 88 years with a mean age of 74.4 years (SD = 7.2 years). For 16%, formal education ended at high school, whereas 12.1% completed college, another 23.1% held a Masters' degree, and an additional 17.3% held a doctorate or medical degree. Regarding income, total annual income for the preceding tax year was reported to be >$45,000 annually by 55.6% with annual incomes of $5,000–15,000, $15,000–25,000, $25,000–35,000, and $35,000–45,000 reported by 7.6, 11.1, 13.5, and 12.3% of the participants, respectively. When queried regarding the duration of their hearing loss, the median response was 5.5 years with an interquartile range of 3–10 years. Regarding current or prior hearing aid use, 33 (19.1%) were currently using hearing aids and 47 (27.2%) reported ever having worn hearing aids with 14 (8.1%) no longer using them.

**Figure 1:**
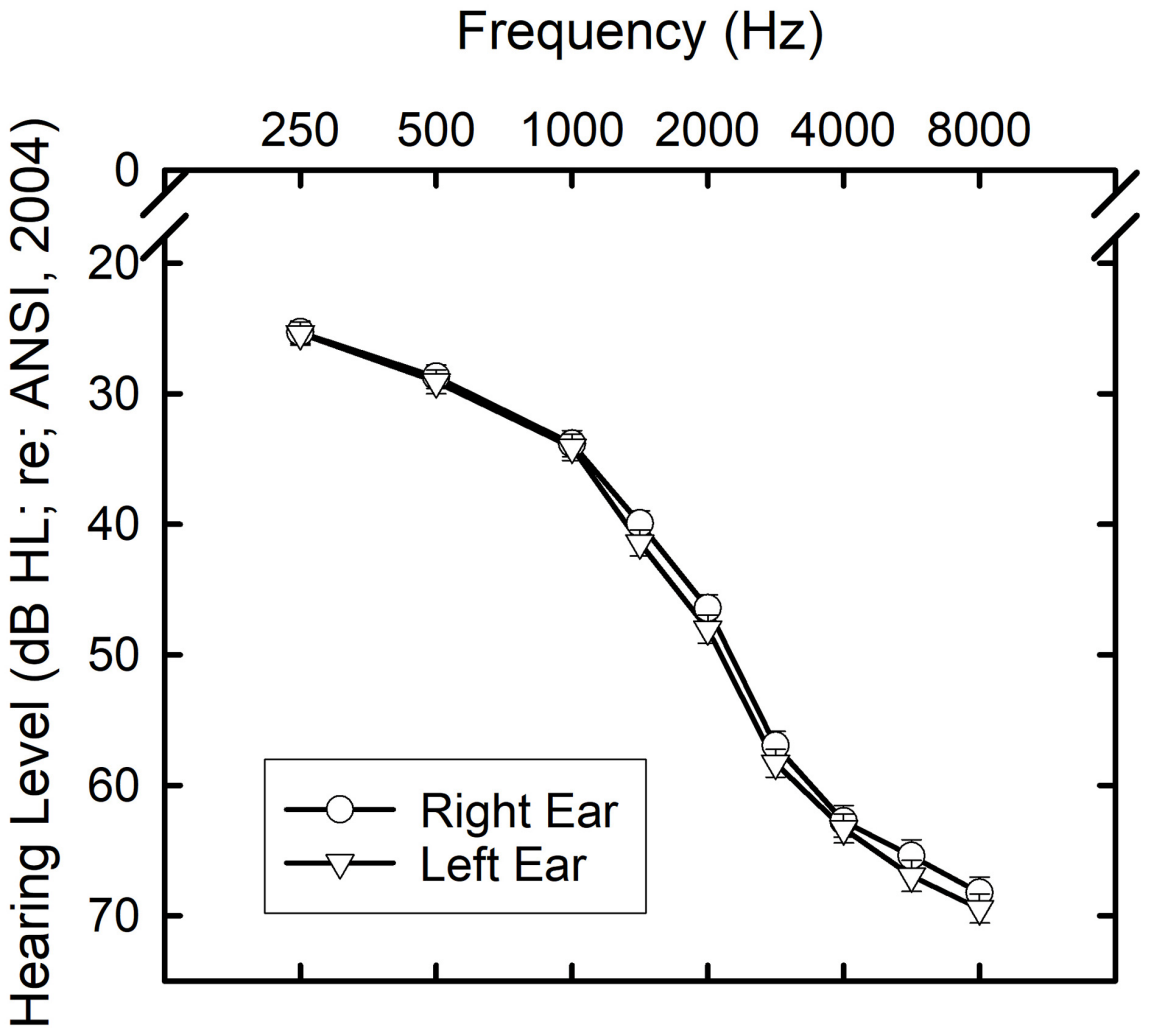
Means and standard errors for the air-conduction thresholds for the right (circles) and left (triangles) ears of the 173 older adults who participated in this study.

Figure 1 provides the mean audiograms for the right and left ears of the 173 older adults. Standard deviations were 11–12 dB at 250 and 500 Hz in each ear and gradually increased with frequency peaking at 14–16 dB at and above 3000 Hz. The audiograms are consistent with those for older adults, showing a bilaterally symmetrical sloping sensorineural hearing loss. Otoscopy, bone-conduction thresholds, and immittance measures confirmed that the hearing loss was sensorineural in nature with no significant conductive components.

Informed consent was obtained from all participants and they were paid on a per-session basis for their participation. This study was approved by the Indiana University Bloomington Institutional Review Board.

### Measures Obtained

In addition to an audiological exam, several other measures pertinent to this study were obtained. These measures were included, in part, to evaluate the validity of the SWB-HL. The measures pertinent to this study could be divided into two categories: (1) psychological measures of affect or stress/anxiety, personality, and cognition; and (2) measures of communication performance and the participant's reaction to difficulties experienced. For the latter, we relied on the Communication Profile of the Hearing Impaired (CPHI; Demorest and Erdman, 1986, 1987). The CPHI represents one of the most comprehensive and rigorously evaluated self-report measures of communication difficulties and the impact of those difficulties on the person's well-being. The 163-item measure is reduced to 25 scales and, ultimately, to five factor scores.

There were three measures of the participant's affect included here. Measures of affect were included to validate the affect components of SWB assessed by the SWB-HL. The Life Orientation Test-Revised (LOT-R; Scheier et al., 1994) is a 10-item measure of optimism vs. pessimism. Three of the 10 items measure either optimism or pessimism with the other four serving as fillers. The participant is presented with a statement and then rates each on a four-point scale of agreement: strongly disagree, disagree, agree, or strongly agree with the three pessimism items reverse scored from the optimism items and the fillers unscored. Earlier work demonstrated the importance of optimism to perceived psychological and physical well-being (Scheier and Carver, 1992).

The Positive and Negative Affect Scale (PANAS), as noted in the introduction, is a brief self-report measure of affect and has been used frequently to capture the two affect components of the tripartite SWB model. The respondent is presented with a list of 20 words in a column and, for each, is asked to indicate whether he or she generally feels this way “on a regular basis” and selects among “very slightly or not at all,” “a little,” “moderately,” “quite a bit” or “extremely” as responses, with points assigned from 1 to 5, respectively. Half of the 20 items convey positive affect, such as “excited,” “strong,” “enthusiastic,” “proud,” and “inspired,” whereas the other have convey negative affect, such as “distressed,” “upset,” “scared,” “ashamed,” and “nervous.” Two scores emerge: PANAS-positive and PANAS-negative. Each represents the total points for each set of 10 items, scores ranging from 10 to 50. For the positive scale, higher scores, and, for the negative scale, lower scores, reflect more positive affect.

The State-Trait Anxiety Inventory (STAI; Spielberger et al., 1970; Spielberger, 1983) is a self-report measure of feelings of anxiety, momentary or in current state and long-term or as a trait of the individual. There are forty items with the first 20 assessing situational or state anxiety, with a focus on how the respondent “feels right now, at this moment,” and the last 20 measuring underlying trait anxiety, how he or she “generally feels.” For all 40 items, four response choices are provided: “not at all,” “somewhat,” “moderately so,” and “very much so” with points of 1, 2, 3, and 4, respectively. Within each scale, several items are reverse scored. Examples from the STAI-S include: “I feel content” and “I am worried.” Examples from the STAI-T are: “I am happy” and “I lack self-confidence.” Total scores, following reversal of some items, range from 20 to 80 for both the STAI-S and STAI-T with higher scores reflecting less anxiety and more positive affect.

In addition, given that other measures of SWB have been demonstrated to be correlated with personality (Steel et al., 2008) and cognitive function (Jones et al., 2003), both types of measures were included here. For personality, the Myers-Briggs Type Inventory (MBTI; Myers et al., 1998) was used and, for cognition, the full Wechsler Adult Intelligence Scale, 3rd Edition (WAIS-III; Wechsler, 1997) was used.

For most of these psychological measures and the CPHI, a Likert-type scale is used to collect participant responses and these responses have been treated like interval data since their inception with norms reported as total scores or mean scores. The assumption has been that the underlying latent constructs tapped by each scale are continuous and the responses represent interval-scale values along that underlying continuum. There has been considerable debate over the years as to whether such Likert-type ratings should be modeled as interval or ordinal data (e.g., Jamieson, 2004; Norman, 2010), but because each of these measures has considered the values to be interval in nature and the norms calculated under this assumption over many decades, the scores from these established scales were treated this way in these analyses as well. For the new SWB-HL scale proposed here, however, this assumption will be examined in greater detail below.

### Procedures

Following the initial case history, otoscopic examination, and audiological assessment, each participant completed a series of measures prior to being fit with amplification. These other measures were completed in separate sessions with the CPHI completed in one, full cognitive assessments with the WAIS-III in another, and the other psychological assessments completed together in a third session. All surveys were completed by the respondent in a pencil-and-paper format without examiner assistance.

After completion of these measures, as well as others not presented here, the participants were then fitted with hearing aids. The technology used varied among one of three options.

One group received four-channel wide-dynamic-range-compression (WDRC) circuits housed in full-concha ITE shells, half with directional microphones and half with omni-directional microphones. The other group received 6-channel open-fit mini-BTE devices with directional microphones. The directional microphones were a fixed super-cardioid configuration, and its function was verified using Verifit software and hardware (Etymonic Design, Dorchester, Canada). Verification of directional performance was obtained in the test box from both hearing aids. The frequency response of the hearing aids programmed for the directional microphone function was equalized to match the frequency response of the hearing aids when set to the omnidirectional-microphone mode. The same basic protocol was used to set and verify target gain for each participant in each group. First, based on audiological information obtained from each participant (air-conduction and bone-conduction hearing thresholds, as well as loudness discomfort levels), target 2-cm^3^-coupler gain values were generated at octave intervals from 250 through 4000 Hz, as well as at 1500, 3000, and 6000 Hz. Hearing aids were adjusted in the 2-cm^3^ coupler for a moderate level input (60–70 dB SPL, across studies) to match target in the coupler and were then fitted to the patient and verified using real-ear probe-tube microphone measurements with adjustments to better match targets performed as needed. The root-mean-square error between target and measured real-ear gain averaged across frequency (250–6000 Hz) was ≤ 5 dB.

The prescriptive procedure used to generate gain targets was NAL-NL1. With each group and technology, software from NAL was used to generate NAL-NL1 targets, rather than the manufacturer's version of that prescriptive protocol. Within a given group of participants, all were fitted bilaterally with identical make and models of hearing aids. In addition, participants paid the typical clinic price for the devices at the time of delivery and then were paid as research subjects for return visits during which they completed a variety of outcome measures.

After on-ear verification of real-ear gain, the participant was counseled about the use, function, and care of the hearing aids. Approximately 4–6 weeks post-fit, the participant returned to complete several outcome measures, with the focus here on the SWB-HL. Reports for several of the conventional outcomes have been published previously for subsets of the study sample reported here with a focus on differences in outcomes for various technologies (Humes et al., 2009, 2010).

## Results and Discussion

### Preliminary Analysis of the SWB-HL Items

As noted, there were initially 18 items in the SWB-HL measure completed by the 173 participants. The responses made use of a 5-point Likert-scale rating for each of the 18 items. There is debate as to whether responses to such items should be modeled as ordinal data or interval data (e.g., Jamieson, 2004; Norman, 2010). Some have demonstrated that, except for extremely skewed distributions of responses, parametric data reduction and analyses of 5-point Likert ratings is appropriate (Flora and Curran, 2004; De Winter and Dodou, 2010). Most recently, Liddell and Kruschke (2018) examined the assumptions regarding the underlying distributions of Likert-type ratings in detail comparing the results for an interval-based metric model to those for an ordered-probit model. They noted that the item means and standard deviations can differ considerably under each model and argue in favor of the ordered-probit model when response distributions show such differences.

Liddell and Kruschke (2018) provided R code to analyze responses from 5-point Likert ratings under both models. This code was used here to generate means and standard deviations from each of the 18 items of the SWB-HL for the two models. Effect sizes were then used to compare those two sets of item means. Although there was a strong correlation between the means under the two model assumptions (*r* = 0.92), the Cohen's d effect sizes (Cohen, 1988) were small for three, medium for two, and large for one of the six items with significant differences in item means. The effect sizes for the difference between the metric and ordered-probit means were all less than small (*d* < 0.2) for the 12 items that were retained. In other words, for the 12 remaining items of the SWB-HL, assumptions about the underlying scale did not impact the item scores and parametric tests could therefore be applied with confidence in the validity of those analyses. In all cases, the six items eliminated had skewed distributions with ordered-probit means ≥ 3.7 (maximum = 4) and skewness values exceeding −1.2, consistent with an extremely skewed distribution associated with a ceiling effect. The 12 items that were retained following this analysis of response distributions were items 1, 2, 3, 4, 5, 8, 11, 13, 15, 16, 17, and 18.

### Factor Analysis of the SWB-HL

Following the paring down of the original 18 items to 12, factor analysis was performed on those 12 items. These analyses made use of the R “psych” package (Version 2.0.12; Revelle, 2020) to facilitate analysis with polychoric correlations. Given the preceding culling of items from the scale, use of parametric Pearson-r correlations for the factor analysis would have likely been acceptable (Flora and Curran, 2004). Nonetheless, non-parametric polychoric correlations optimized for the analysis of Likert-type responses were instead computed for these 12 items and the ensuing factor analysis was based on this matrix of polychoric correlations. The analysis of the SWB-HL began with an exploratory principal-axis factor analysis (Gorsuch, 1983) of the responses to the 12 survey items. Exploratory factor analysis was chosen because this was the first evaluation of the SWB-HL. Thus, no apriori assumptions were made about underlying scales, if any, with this to be determined by the results of the exploratory factor analysis. Initially, oblique rotation of factors was considered which allows for correlations among the factors that emerge. Three factors emerged and all resulting between-factor correlations were < 0.35. As a result of these weak inter-factor correlations, a second principal-axis factor analysis was performed, this time with orthogonal (varimax) rotation of factors resulting in three independent factors. A reasonable fit was obtained, accounting for 58% of the variance, but two items had low communalities (0.22, 0.33) indicating that neither was well-represented in the three-factor solution. These two items, item 11 and 16, were deleted. The remaining 10 items were analyzed again with the same principal-axis factor analysis with varimax rotation and a three-factor solution again emerged, accounting for 65% of the variance and all communalities ≥0.43. Each of the three rotated factors accounted for nearly equivalent amounts of variance. The root-mean-square of the residuals for this final factor solution for the SWB-HL was 0.05 with a Bayesian Information Criterion of 25.4, both reflecting a good fit of the solution to the data. Finally, a hierarchical factor analysis was performed using the *omega* function in the R psych package to determine whether all 10 items were represented by a general (g) underlying factor (Revelle, 2020). This would better validate the use of a total score for all 10 items as a measure representing this general factor common to all items.

**Figure 2:**
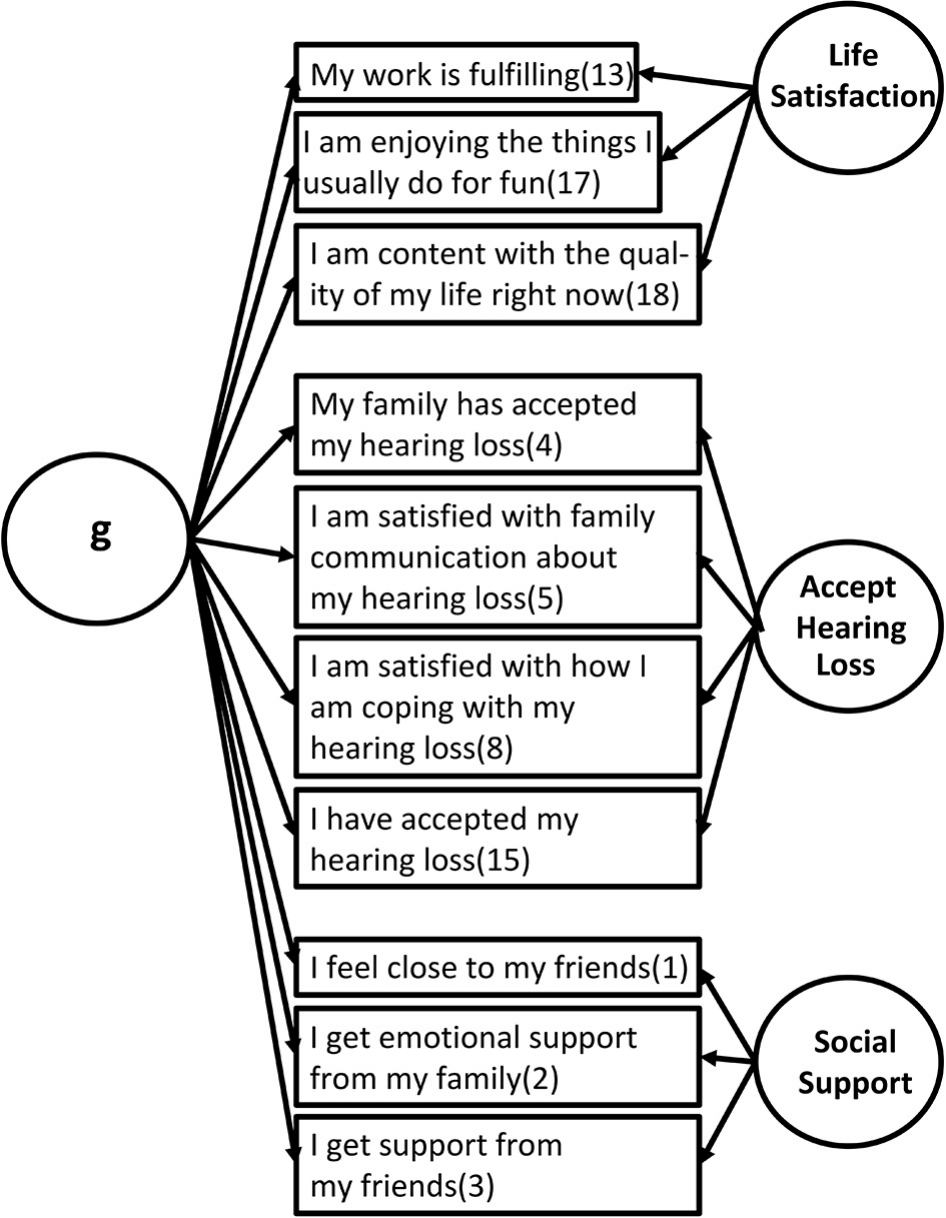
Hierarchical factor structure for the 10 items of the SWB-HL scale showing the association of each item to the factors identified. The number in parentheses after each item is the item number in the original 18-item corpus.

Figure 2 graphically depicts the results of the hierarchical factor analysis and Table 1 provides the rotated factor loadings from the pattern matrix of the hierarchical solution for each of the 10 SWB-HL items. As can be seen, all 10 items are weighted both on the general factor (g) and one of the three domain-specific factors. Based on the factor loadings in Table 1, the three scale factors were interpreted as Life Satisfaction, Acceptance of Hearing Loss, and Social Support. The three factors accounted for 23, 21, and 21% of the variance, respectively, for the rotated solution.

**Table 1:** Rotated factor loadings for the each of the 10 SWB-HL items (principal-axis factor analysis, Oblimin rotation) on the three resulting factors and the general factor, g.

	**g**	**AccHL**	**SocSupp**	**LifeSat**
1. I feel close to my friends.	0.35		0.77	
2. I get emotional support from my family.	0.39		0.57	
3. I get support from my friends.	0.44		0.77	
4. My family has accepted my hearing loss.	0.50	0.64		
5. I am satisfied with family communication about my hearing loss.	0.58	0.74		
8. I am satisfied with how I am coping with my hearing loss.	0.49	0.38		
13. My work (include work at home) is fulfilling.	0.49			0.50
15. I have accepted my hearing loss.	0.47	0.38		
17. I am enjoying the things I usually do for fun.	0.62			0.55
18. I am content with the quality of my life right now.	0.62			0.61
6. I feel close to my partner (or my main support).				
7. I feel sad.				
9. I am losing hope in the fight against my hearing loss.				
10. I feel nervous.				
11. I worry that my hearing loss will get worse.				
12. I am able to work (include work at home).				
14. I am able to enjoy life.				
16. I am sleeping well.				

*Only factor loadings > 0.25 are shown for clarity. Items highlighted in gray at the bottom of the table were deleted from the original corpus of 18 items prior to this analysis and are shown for completeness (see text for details.). These factor loadings can be linked to the arrows in Figure 2 to show the strength of association of each item to the various factors*.

These analyses suggest that the SWB-HL taps three separate aspects of SWB in older adults with hearing loss. The analyses also suggest that the SWB-HL Total score taps a general underlying construct common to all three factors.

### Reliability of the SWB-HL

The SWB-HL could be used to generate four scores based on the general factor, g, and each of the three domain-specific factors. Although the analyses of the SWB-HL support the existence of three domains captured by the 10-item scale, it is not recommended that such individual subscale scores be used due to the small number of items, 3–4, comprising each scale. Instead, it is recommended that just the SWB-HL Total be used with the knowledge that it is tapping the constructs of Life Satisfaction, Acceptance of Hearing Loss, and Social Support. The full scale, 10 items, is itself brief and it is well-known that the reliability of any measure decreases as the number of items comprising the measure decrease. Moreover, the correlations of the SWB-HL Total score with the three subscale scores were *r* = 0.71, 0.67, and 0.84, all significant (*p* < 0.01), for the Life Satisfaction, Social Support, and Acceptance of Hearing Loss scales, respectively. This again reinforces that a single 10-item SWB-HL Total score will capture variations in each of these three domains.

With this in mind, reliability analyses included the computation of Cronbach's alpha (Cronbach, 1951) across all scale items, computation of the mean inter-item correlation (r_ii_), and examination of the change in Cronbach's alpha following deletion of each item. The reliability analyses were completed using the *alpha* function of the R psych package and the polychoric correlation matrix for the 10 items. Cronbach's alpha for the SWB-HL Total score was 0.85 (95% confidence interval, 0.82–0.89) showing strong internal consistency of the measure. In general, the desired range for the mean inter-item correlation is 0.15 < r_ii_ < 0.50 (Cronbach, 1984). The mean inter-item correlation for the 10 SWB-HL Total items ranged from 0.35 to 0.38, well within the desired range. Finally, Cronbach's alpha was recomputed several times after each item of the scale was deleted. This is a way to determine if the internal consistency of the scale would be increased by removing a particular item (Cronbach, 1984). For the SWB-HL Total score, Cronbach's alpha failed to increase above 0.85 after removal of any of the 10 items from the scale. Based on these analyses it is concluded that the 10-item SWB-HL Total score is reliable.

### Validity of the SWB-HL

In addition to completing the SWB-HL, as noted above, all participants also completed the full 163-item CPHI, several measures of affect, a personality scale, and a cognitive assessment. It is expected, if the SWB-HL is a valid measure of SWB, that there would be some associations between the SWB-HL and these other measures. This type of validity is generally referred to as criterion validity and is a common approach to validation of self-report measures (Cronbach, 1984). Basically, if the SWB-HL is a valid measure of SWB it would be expected to be related to measures found previously to be associated with SWB.

Linear multiple-regression analysis was used to examine the associations between SWB-HL Total scores and these other measures. First, however, the number of criterion variables was reduced through principal-components factor analysis (varimax rotation). It should be noted that, although several of these scales make use of Likert-type responses, factor analysis does not appear to be impacted by assumptions of ordinal or interval data except for extremely skewed ordinal distributions (Flora and Curran, 2004). When the 23 CPHI scale scores were analyzed, a good fit was obtained and six factors emerged, accounting for 76% of the variance and all communalities >0.62. Four of the six factors matched four of the five factors reported by Demorest and Erdman (1989) for a similar analysis of the CPHI, corresponding to factors of Personal Adjustment (PA), Communication Performance (CP), Communication Importance (CI), and Interactions with Others (Interax). The remaining factor from Demorest and Erdman (1989), Reaction to Hearing Loss, was split between two factors here: Communication Environment, need and physical characteristics (React1); and Communication Strategies, verbal and non-verbal (React2). Of the three datasets analyzed by Demorest and Erdman (1989), one of the three showed a similar 6-factor solution with a split of the Reaction factor along the same lines as observed here. It is concluded that the CPHI factors identified here are consistent with those identified originally by Demorest and Erdman (1989).

When the five measures of affect were subjected to a similar principal-components analysis a single factor emerged accounting for 58% of the variance and four of the five communalities were >0.49. Only the PANAS-positive score had a somewhat lower communality, 0.32, indicating that performance on it may not be quite as well-represented by the single factor score than the other four measures of affect. When the factor loadings for each of the five affect measures on the single factor were examined, negative weights were observed for the two positive measures, LOT-R and PANAS-positive, and positive weights for the three negative affect scores, PANAS-negative, STAI-state, STAI-trait. Thus, higher factors scores reflect more negative affect. The affect factor score is referred to here as PC_Affect.

Finally, similarly good fits from principal-components factor analysis were obtained with four orthogonal factor scores emerging for the Myers-Briggs Type Inventory (MBTI) personality scale (Myers et al., 1998) and three orthogonal factor scores from the full 13-scale Wechsler Adult Intelligence Scale-3rd Edition (Wechsler, 1997: WAIS-III) cognitive measure. The four personality factors represented the dimensions of introversion/extraversion, perceiving/judging, thinking/feeling, and intuition/sensing at the core of the MBTI personality types. For the WAIS-III, the three factors represented the four major types of cognitive processing identified by the test, verbal comprehension (VC), working memory (WM), perceptual organization (PO), and processing speed (PS), with the latter two combined into a single factor.

A linear multiple-regression analysis was next conducted with the SWB-HL Total score as the dependent measure. The SWB-HL Total score was z-transformed prior to regression analysis. In addition to the orthogonal factor scores for the CPHI, Affect, MBTI, and WAIS-III, all of which have a mean of 0 and standard deviation of 1, age and better-ear four-frequency pure-tone average (PTA4 BtrE) were z-transformed and added as additional independent variables to each regression equation.

Table 2 provides the results of the regression analyses for the SWB-HL Total score. Predictors with significant (*p* < 0.05) Beta coefficients in the standardized regression equation are highlighted in bold font and the associated zero-order, partial, and part correlations are italicized for those significant contributors. Five significant predictors emerged for the SWB-HL Total score with the solution accounting for 48% of the variance (r^2^). In addition, collinearity among the predictors was not an issue with the Variance Inflation Factor (VIF) < 1.62 and the Condition Index < 2.7 for all predictors.

**Table 2:** Results of linear multiple-regression analyses for the SWB-HL Total.

	**Beta**	***t***	***p***	**Zero-order r**	**Partial r**	**Part r**
(Constant)		103.234	0.000			
Zscore: AGE	0.051	0.709	0.479	0.053	0.057	0.041
Zscore (PTA4 BtrE)	−0.115	−1.565	0.120	−0.217	−0.124	−0.090
**PC_Affect**	**−0.342**	**−4.753**	**0.000**	*−0.548*	*−0.355*	*−0.275*
**PC_CPHI_PA**	**0.211**	**3.110**	**0.002**	*0.388*	*0.241*	*0.180*
PC_CPHI_CP	0.039	0.578	0.564	0.116	0.046	0.033
PC_CPHI_CI	0.096	1.512	0.132	0.173	0.120	0.087
**PC_CPHI_Interax**	**0.182**	**2.880**	**0.005**	*0.293*	*0.224*	*0.166*
PC_CPHI_React2	0.081	1.316	0.190	0.061	0.104	0.076
PC_CPHI_React1	0.050	0.808	0.420	−0.012	0.064	0.047
PC MBTIsense_intuit	0.064	0.908	0.365	0.123	0.072	0.052
PC MBTIperceiv_judge	0.097	1.626	0.106	0.185	0.129	0.094
**PC MBTIthink_feel**	**0.169**	**2.751**	**0.007**	*0.177*	*0.214*	*0.159*
**PC MBTIintrov_extrav**	**0.123**	**1.981**	**0.049**	*0.235*	*0.156*	*0.114*
PC W3_VC	−0.075	−1.045	0.298	0.086	−0.083	−0.060
PC W3_PS_PO	−0.042	−0.599	0.550	−0.024	−0.048	−0.035
PC W3_WM	0.102	1.640	0.103	0.212	0.130	0.095

*Significant Beta regression coefficients are shown as p-values in bold and r values in italics. Dependent Variable: SWB-HL Total (r^2^ = 0.48; [F_(16,157)_ = 8.93], p < 0.001). PTA4 BtrE, pure-tone average for 500, 1000, 2000, and 4000 Hz in the better ear; PC, Principal Component; CPHI, Communication Profile of the Hearing Impaired; PA, Personal Adjustment; CP, Communication Performance; CI, Communication Importance; Interax, Interactions with others; React1 and React2 are two components regarding the individual's reaction to hearing impairment; MBTI, Myers-Briggs Type Inventory; W3, Wechsler Adult Intelligence Scale, 3rd Edition; VC, Verbal Comprehension; PS PO, Processing Speed/Perceptual Organization; WM, Working Memory*.

The linear multiple-regression analysis summarized in Table 2 revealed significant associations between various SWB-HL scores and other criterion measures with the pattern reflecting expected associations. Of the five significant predictors in Table 2, two were captured by aspects of the CPHI; CPHI PA, a factor related to personal adjustment to hearing loss, and CPHI Interax, a factor representing communication interactions with others. It is not surprising that the CPHI PA scale emerged as a prominent predictor of SWB-HL as this measure includes assessment of self-acceptance, stress, anger, and withdrawal, among others, and would clearly impact one's self-reported SWB (Steel et al., 2008). Similarly, CPHI Interax would likely be tied closely to the social-support items of the SWB-HL scale and social support has a positive impact on SWB (Diener and Seligman, 2002; Siedlecki et al., 2014). Two other significant predictors identified were personality measures, the extraversion/introversion and thinking/feeling dimensions of the MBTI. Personality has long been known to influence general SWB, with consistent ties to extraversion and neuroticism (e.g., Steel et al., 2008; Strickhouser et al., 2017). The MBTI generally does not capture neuroticism (McCrae and Costa, 1989), but many neurotic personality characteristics are captured by several of the CPHI-PA scales as noted above. Finally, the affect factor score was the single-best predictor of SWB-HL Total scores. This reflects the long-time recognition of the strong association between affect and life-satisfaction measures of SWB (Diener, 1984; Diener et al., 1999) as well as the link between affect and hearing-loss-related quality of life (Preminger and Meeks, 2010).

Perhaps as interesting are the factors that proved not to be associated with SWB-Total performance. These include the severity of hearing loss, age, and cognition. Associations between cognitive function and general SWB have been observed previously (e.g., Jones et al., 2003; Siedlecki et al., 2020) but these associations may be mediated by several other factors (Yazdani and Siedlecki, 2020). Although the age range included here was restricted to older adults and no effect of age over this range of 60–88 years was observed here, SWB has been found to be relatively stable over the adult lifespan (Stone et al., 2010; Braun et al., 2017). Sensory loss, including hearing loss, has been associated with poorer SWB (Ciorba et al., 2012; Tseng et al., 2018) but no effects were observed here. A likely reason for the failure of hearing loss severity to emerge as a significant predictor in these analyses may be found in the limited range of hearing loss in this sample (Figure 1). This homogeneity of hearing loss severity may, in turn, have contributed to the inability of the CPHI Communication Performance (CP) and Communication Importance (CI) scales to predict SWB-HL Total scores. Regression analyses depend on sufficient variation in independent and dependent variables to identify significant predictors and the homogeneity of hearing loss and perceived communication performance among this sample may have impacted the results of those analyses. Nonetheless, except for the absence of the impact of hearing loss, those measures found to be significant predictors of SWB-HL Total scores, as well as those identified as not being predictive of performance, reveal a pattern consistent with the expectations for a general measure of SWB primed by probes of hearing loss.

### Aided and Unaided SWB-HL Scores

Another form of validation which focuses on the sensitivity of the instrument is to examine the scores before and after intervention (Ventry and Weinstein, 1983). As noted in Methods, 143 of the 173 participants were fitted with hearing aids and of these 143, 141 had complete pre-fit and post-fit SWB-HL data. The mean SWB-HL Total score was 31.32 (SD = 5.16) prior to the hearing-aid fit and 32.84 (SD = 5.07) after 4–6 weeks of hearing aid use. A paired-sample *t*-test showed this difference to be significant [*t*_(140)_ = 4.29, *p* < 0.001] with a medium effect size (Cohen's *d* = 0.36, 95% CI = 0.19–0.53). Significant improvements in SWB were observed following 4–6 weeks of hearing-aid use with the SWB-HL Total.

Perhaps the aided improvement in SWB-HL Total score is carried exclusively by the four items of the SWB-HL in the Acceptance of Hearing Loss domain. This pattern would be consistent with the prior literature reviewed in the Introduction suggesting that only domain-specific improvements are expected in SWB measures. Given that four of the 10 items in the SWB-HL Total score make queries about the impact of hearing loss perhaps aided improvements of these four items are sufficient to result in the observed improvement in SWB-HL Total score with amplification. To examine this, a 6-item SWB score was computed from the three Life Satisfaction and the three Social Support items. These non-HL SWB scores were then compared for the pre-fit and post-fit measurements. The mean SWB-HL Total “non-hearing loss” score was 19.45 (SD = 3.17) prior to the hearing-aid fit and 19.93 (SD = 3.28) after 4–6 weeks of hearing aid use. A paired-sample *t*-test showed this difference to be significant [*t*_(140)_ = 2.33, *p* < 0.05] but with a small effect size (Cohen's *d* = 0.20, 95% CI = 0.03–0.36). When just the four items tapping Acceptance of Hearing Loss were totaled for evaluation pre- and post-fit, the post-fit mean (12.91, SD = 2.57) was significantly greater [*t*_(140)_ = 4.35, *p* < 0.001] than the pre-fit mean (11.87, SD = 3.08) and a medium effect size was observed (*d* = 0.37). Given that the hearing aid intervention addresses the hearing loss of the respondent, and consistent with the prior literature on the demonstration of domain-specific improvements in SWB following hearing-aid use, the increased score for SWB-HL items tapping Acceptance of Hearing Loss is expected. More importantly, the increased score for the 6-item SWB-HL score calculated for the general SWB items reflects broader improvements in SWB from amplification in older adults with hearing loss. Again, such subscale scores of the SWB-HL are not being recommended here for use but as a means to demonstrate that hearing aid use impacted more than just the hearing-related items of the SWB-HL.

**Figure 3:**
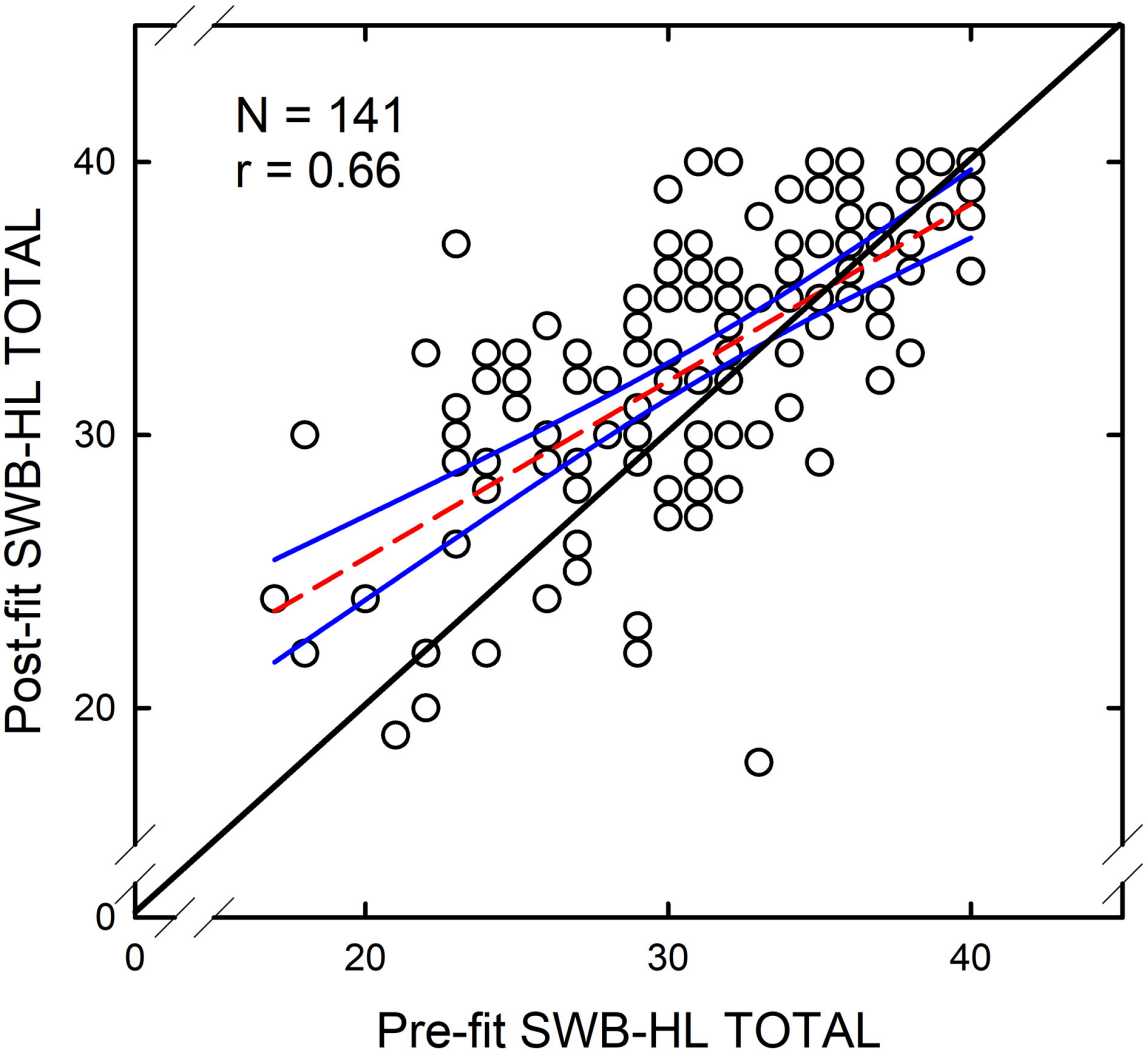
Scatterplot of aided SWB-HL Total scores against unaided SWB-HL Total scores for 141 older adults fitted with hearing aids in this study. The correlation between these two scores was *r* = 0.66 (*p* < 0.001). Each circle represents an individual data point. The solid black line along the diagonal represents equality of the aided and unaided scores. The dashed red line is the best-fitting linear-regression equation and the blue lines represent 95% confidence intervals around that equation.

The Pearson-r correlation between pre-fit and post-fit SWB-HL Total scores was 0.66 and statistically significant (*p* < 0.001). This correlation, coupled with the increase in mean performance for the group, suggest that the general trend across individuals was for the use of hearing aids for this 4–6-week period to improve the measured SWB. Figure 3 shows a scatterplot of the individual unaided and aided SWB-HL Total scores. The best-fitting linear regression equation is plotted in this figure as a dashed red line with 95% confidence intervals around this best-fitting line shown as blue solid lines. As can be seen, those with lower SWB pre-fit showed the largest improvements in SWB post-fit. Generally, if pre-fit SWB was good, SWB-HL Total > 30, then improvements in SWB with hearing aids were smaller and less frequent. Twenty-five percent of the 141 individuals showed an improvement of four points or more in the SWB-HL Total score which can range from 0 to 40.

As noted previously, of the 141 individuals with complete SWB-HL unaided and aided scores, 43 had worn hearing aids at some point in the past and 30 of those 43 were current hearing aid users when they enrolled in this study. As a result, assuming a positive impact of hearing aids on SWB, those with prior hearing aid use may have had higher pre-fit scores and would have shown less improvement in SWB over the 4–6-week course of this study. To determine whether inclusion of those with prior hearing aid experience impacted the analyses of pre- and post-fit SWB-HL Total scores, the paired-sample *t*-tests were again performed for the 98 who had never worn hearing aids and the 111 who were not currently using hearing aids. The pattern of significant differences described previously for the entire group of 141 was the same for the subgroups of 98 and 111 older adults with prior hearing aid experience. The same is true for the magnitude and significance of the correlations between the post-fit and pre-fit SWB-HL Total scores as well as the Cohen's d effect sizes for the aided improvements in SWB-HL Total.

Clearly, based on the data and correlation in Figure 3, the best predictor of *aided* SWB-HL scores is most likely the *unaided* SWB-HL score. To examine this further, the multiple linear-regression analysis conducted for the pre-fit SWB-HL Total scores was repeated for the post-fit scores but with the addition of the pre-fit SWB-HL and a hearing-aid expectations factor representing the four scales of the Expected Consequences of Hearing Aid Ownership (ECHO; Cox and Alexander, 2000). SWB-HL Total scores, pre-fit and post-fit, were converted to z-scores for these analyses. Conversion of several measures to z-scores matched the means and standard deviations of these z-transformed variables to those of the various factor scores used as predictors such that all variables in the analyses had means of 0 and standard deviations of 1.

With the z-transformed post-fit (aided) SWB-HL-Total score as the dependent measure, the best-fitting regression solution accounted for 50.8% of the variance but only had one significant predictor variable, the z-transformed pre-fit (unaided) SWB-HL Total score. Thus, these analyses with the inclusion of a broad range of potential predictors confirm the relationship between pre-fit and post-fit SWB-HL-Total scores illustrated previously in Figure 3. Aided SWB is largely determined by the pre-fit unaided SWB.

If the unaided SWB-HL Total score is not included among the independent measures and the regression analysis predicting aided SWB-HL Total score is repeated, only two significant predictors emerged: affect (*r* = −0.42) and ECHO (*r* = 0.29). This solution, however, only accounted for 34% of the variance compared to nearly 51% when unaided SWB-HL scores were included. The fact that neither affect or ECHO were significant predictors when the unaided SWB-HL total scores were included implies that the unaided SWB-HL Total score captures aspects of the individual's self-reported affect and hearing-aid expectations.

As demonstrated above, pre-fit, unaided SWB, as captured by the SWB-HL Total score, was largely determined by personality, affect, and the individual's adjustment to hearing loss, both in terms of the individual's affect and his or her interactions with others. Of course, personality, is a factor that would not be considered malleable by the audiologist to improve pre-fit and, consequently, post-fit SWB. The CPHI measures, on the other hand, can be shaped by the clinician through aural rehabilitation and counseling. The CPHI, in fact, was developed as an assessment tool to determine the focus of subsequent aural rehabilitation (Demorest and Erdman, 1986, 1987). Affect, both generally and as impacted by hearing loss, is also something that could be modified potentially through counseling. Whicker et al. (2020) recently noted associations between various psychological measures and SWB and encouraged audiologists to take a more active role in shaping the thoughts and emotions of patients regarding their hearing loss. The present findings suggest that doing so will enhance SWB, both unaided and aided, at least as measured by the SWB-HL.

In summary, the foregoing analyses of SWB-HL scale scores for unaided and aided listening conditions further validate this measure. When amplification was fit to these older adults, the SWB-HL Total score demonstrated sufficient sensitivity to support improved SWB after the 4–6-week hearing-aid trial. Regression analyses identified areas of focus for rehabilitation that may lead to enhanced SWB with amplification in older adults.

## Conclusions

The final 10-item version of the SWB-HL yielded a total score linked to the constructs of Life Satisfaction, Social Support, and Acceptance of Hearing Loss. This report documented the reliability and validity of the SWB-HL Total score. Generally, the greater the number of items comprising a test, the more reliable the result. As such, use of the SWB-HL Total score is recommended, although it is possible to get more specific information about SWB in older adults with hearing loss by examining the individual subscales. Regarding validity, the SWB-HL Total score was associated with criterion measures administered separately, especially the measures of Personal Adjustment from the CPHI, personality, and affect. Thus, the 10-item SWB-HL Total score captures both general and hearing-loss-specific components of SWB. Further demonstration of the validity of the 10-item SWB-HL Total was demonstrated through significant differences and medium effect sizes for comparisons before and after hearing-aid use. It is noteworthy that such differences in SWB were measurable following a 4–6-week period of hearing aid use. Regression analyses resulted in a potential model of the key determiners of SWB in older adults with hearing loss. This model requires further evaluation and validation.

A limitation of this study is the restricted nature of the study sample, both in terms of size and demographic characteristics (e.g., white, well-educated, and reasonable annual income). In addition, we noted the importance of item context in the Introduction, including the potential impact of priming. The final 10 items comprising the SWB-HL were extracted from the initial larger set of 18 items. It is unknown how the eight items that have been eliminated from the original corpus of 18 may have impacted the responses for the 10 items that remained. Further research is needed to overcome these limitations. In addition, these context effects should be kept in mind by developers of future questionnaires or surveys in the field.

## Data Availability Statement

The raw data supporting the conclusions of this article will be made available by the authors, without undue reservation.

## Ethics Statement

The studies involving human participants were reviewed and approved by Indiana University Bloomington IRB. The patients/participants provided their written informed consent to participate in this study.

## Author Contributions

LH performed all the data analyses described here and wrote the manuscript without assistance.

## Conflict of Interest

The author declares that the research was conducted in the absence of any commercial or financial relationships that could be construed as a potential conflict of interest.
