# Supplementary material for: Development of the SWB-HL: A Scale of the Subjective Well-Being of Older Adults With Hearing Loss
Source: Front Psychol. 2021 Jun 11;12:640165. doi: 10.3389/fpsyg.2021.640165 (PMC8226080; doi:10.3389/fpsyg.2021.640165)
Supplement: Supplementary file 1 [file Data_Sheet_1.PDF]

Name: ________________________________ **Date:**_______________

SWB-HL

INSTRUCTIONS: Below is a list of statements that other people with hearing loss have said are important. **By checking one (1) of the five choices available, please indicate how true each statement has been for you during the past 7 days**.

1. I feel close to my friends.

CHECK ONE:
- ☐ Not at all
- ☐ A little bit
- ☐ Somewhat
- ☐ Quite a bit
- ☐ Very much

2. I get emotional support from my family.

CHECK ONE:
- ☐ Not at all
- ☐ A little bit
- ☐ Somewhat
- ☐ Quite a bit
- ☐ Very much

3. I get support from my friends.

CHECK ONE:
- ☐ Not at all
- ☐ A little bit
- ☐ Somewhat
- ☐ Quite a bit
- ☐ Very much

4. My family has accepted my hearing loss.

CHECK ONE:
- ☐ Not at all
- ☐ A little bit
- ☐ Somewhat
- ☐ Quite a bit
- ☐ Very much

5. I am satisfied with family communication about my hearing loss.

CHECK ONE:
- ☐ Not at all
- ☐ A little bit
- ☐ Somewhat
- ☐ Quite a bit
- ☐ Very much

⇨

INSTRUCTIONS: Below is a list of statements that other people with hearing loss have said are important. **By checking one (1) of the five choices available, please indicate how true each statement has been for you during the past 7 days**.

6. I am satisfied with how I am coping with my hearing loss.

CHECK ONE:

- ☐ Not at all
- ☐ A little bit
- ☐ Somewhat
- ☐ Quite a bit
- ☐ Very much

7. My work (include work at home) is fulfilling.

CHECK ONE:

- ☐ Not at all
- ☐ A little bit
- ☐ Somewhat
- ☐ Quite a bit
- ☐ Very much

8. I have accepted my hearing loss.

CHECK ONE:

- ☐ Not at all
- ☐ A little bit
- ☐ Somewhat
- ☐ Quite a bit
- ☐ Very much

9. I am enjoying the things I usually do for fun.

CHECK ONE:

- ☐ Not at all
- ☐ A little bit
- ☐ Somewhat
- ☐ Quite a bit
- ☐ Very much

10. I am content with the quality of my life right now.

CHECK ONE:

- ☐ Not at all
- ☐ A little bit
- ☐ Somewhat
- ☐ Quite a bit
- ☐ Very much

END
