# Supplementary material for: Development of the SWB-HL: A Scale of the Subjective Well-Being of Older Adults With Hearing Loss
Source: Front Psychol. 2021 Jun 11;12:640165. doi: 10.3389/fpsyg.2021.640165 (PMC8226080; doi:10.3389/fpsyg.2021.640165)
Supplement: Supplementary file 2 [file Data_Sheet_2.PDF]

Subjective Well-Being of Older Adults with Hearing Loss (SWB-HL)

Scoring Instructions

Points are assigned to each response as follows:

Not at all	0
A little bit	1
Somewhat	2
Quite a bit	3
Very much	4

Scale scores:

Acceptance of Hearing Loss: Total points for items 4, 5, 6 and 8.

Social Support: Total points for items 1, 2 and 3.

Life Satisfaction: Total points for items 7, 9 and 10.

SWB-HL Total score, *the recommended 10-item score*, is the sum for items 1-10.
